# Protocol for editing fibroblasts with *in vitro* transcribed Cas9 mRNA and profile off-target editing by optimized GUIDE-seq

**DOI:** 10.1016/j.xpro.2023.102662

**Published:** 2023-10-27

**Authors:** Zhuokun Li, Ganna Reint, Emma Maria Haapaniemi

**Affiliations:** 1 Centre for Molecular Medicine Norway, University of Oslo, Oslo, Norway

**Keywords:** Cell Culture, CRISPR, Sequencing

## Abstract

CRISPR-Cas9 gene editing is an efficient technique to modify specific sites/regions of DNA. Delivery of the Cas9 by mRNA is particularly promising in pre-clinical genome editing applications for its transient, nonintegrating feature. However, the off-target of Cas9-gRNA still remains a concern and needs a specific monitor. Here, we present a revised protocol to edit fibroblasts by *in vitro* transcribed Cas9 mRNA and profile its off-target effect by the optimized GUIDE-seq method. This protocol can also be applied to other cell lines.

For complete details on the use and execution of this protocol, please refer to Ganna Reint et al. (2021).^1^

## Highlights

• A protocol for *in vitro* transcription of Cas9 mRNA with quality control step
• Electroporation transfection to edit fibroblast with Cas9 mRNA
• Optimization of dsODN concentration used for GUIDE-seq
• Profile off-target editing by optimized GUIDE-seq method

## Abstract (editor highlights)

Publisher’s note: Undertaking any experimental protocol requires adherence to local institutional guidelines for laboratory safety and ethics.

## Graphical abstract

**Figure:**
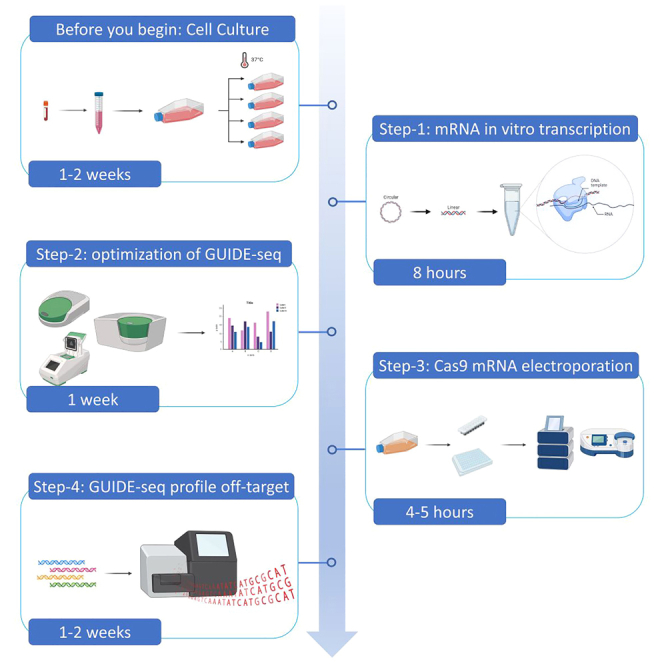

## Before you begin

The protocol below describes the specific steps for using human primary fibroblast cells. And we have also used this protocol in human peripheral blood mononuclear cells (PBMCs).

**Figure 1:**
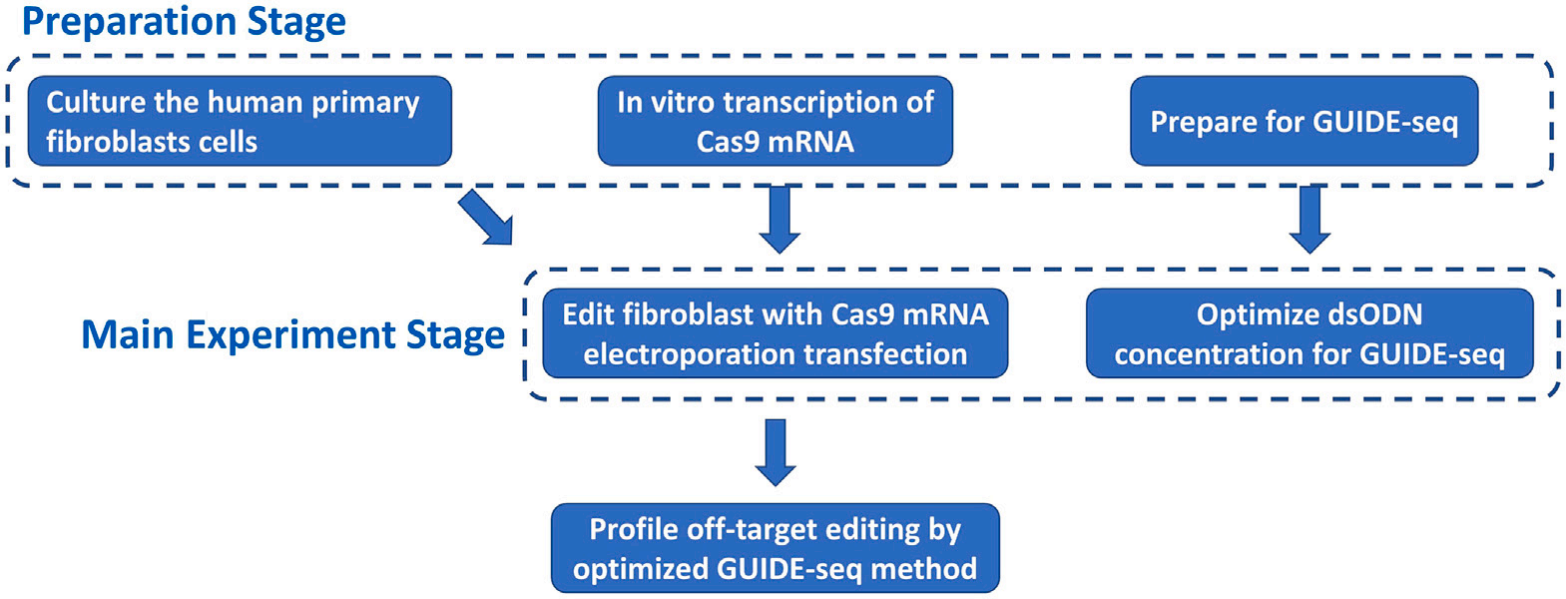
Overview and flow-chart of this protocol

***Note:*** This protocol can be divided into the preparation stage and main experiment stage as the following flow chart indicates (Figure 1). Even though the “*in vitro* transcription of Cas9 mRNA” belongs to the preparation stage, it involves several steps and is critical to the success of the main experiment, we described it elaborately in this protocol.

### Culture the human primary fibroblasts cells

**Timing: 1–2 weeks**

***Note:*** Culture conditions may differ between cell types. Please follow your established procedure or the supplier’s recommendations. Do not use cells after passage 9 for electroporation. Cells should be passaged 3–5 days before electroporation. Optimal confluency before electroporation: 75%–80%.

1. Cell culture recommendations before electroporation.
  a. Seeding conditions: 2–6 × 10^4^ cells/cm^2^.
  b. Replace medium every 2 days until cells reaches about 70%–85% confluency.
  c. Cells should be passaged after reaching 90% confluency; higher confluency may reduce viability of the cells.
2. Trypsinization.
  a. Remove media from the cultured cells and wash cells once with PBS; use at least same volume of PBS as culture media.
  b. Incubate the cells 5 min at 37°C with recommended volume of Trypsin-EDTA (0.05%).
  c. Neutralize trypsinization reaction with full cell culture medium containing FBS once the majority of the cells (>90%) have been detached (no more than 10 min as otherwise cells may start to clump). The volume of the full cell culture medium depends on the size of the cell culture dishes or flasks, which can be found in the link provide below.
3. Split the cells to required number of T-175 flasks to obtain 75%–80% confluence in 48 h. Use the full cell culture medium (35 mL per T-175 flask). The cell number seeded can be found in recommendation from step 1 or from the link below.

***Note:*** for using other sizes of dishes and flasks for cell culture, the numbers, such as cell seeding density, volume of culture medium and trypsin reagent, can be found from this link: https://www.thermofisher.com/no/en/home/references/gibco-cell-culture-basics/cell-culture-protocols/cell-culture-useful-numbers.html

### Preparation Y-adapter for GUIDE-seq

**Timing: 1 h**

4. The Y-adapter is made by annealing the Miseq common oligo with each of the sample barcode adapters (A01 to A16, see key resources table). The adapters also contain 8-mer NNWNNWNN (N = A, C, T, or G; W = A or T) molecular indexes.

**Table:** 

Reagent	Amount
1× TE Buffer	80.0 μL
A## (100μM)	10.0 μL
MiSeq Common Adapter_MI (100μM)	10.0 μL
**Total**	**100.0 μL**

5. Set the Annealing program on thermocycler machine: 95°C for 1 s; 60°C for 1s; slow ramp down (approximately −2°C/min) to 4°C, then hold at 4°C.
6. Store the annealed Y-adapter in −20°C. Annealed Y-adapters can be stored at −20°C for at least one year, we have not performed longer tests.

## Key resources table

**Table:** 

REAGENT or RESOURCE	SOURCE	IDENTIFIER
**Chemicals, peptides, and recombinant proteins**		

Alt-R S.p. Cas9 Nuclease V3, 500 μg	IDT	1081059
KCl	Sigma-Aldrich	P9541
MgCl_2_ × 6H_2_O	VWR	#2510.295
Na_2_HPO_4_ × 7H_2_O	Sigma-Aldrich	#S9390
NaH_2_PO_4_ × 2H_2_O	Sigma-Aldrich	# 04269
D-mannitol	Sigma-Aldrich	#M4125

**Critical commercial assays**		

HiScribe T7 ARCA mRNA Kit (with tailing)	NEB	E2060S
P3 Primary Cell 4D-NucleofectorTM X Kit S	Lonza	V4XP-3032
P3 Primary Cell 96-well Kit (96 RCT)	Lonza	V4SP-3096
Lithium chloride precipitation solution	Thermo Fisher	AM9480
Nuclease-free water	Thermo Fisher	AM9937
Formaldehyde load dye	Invitrogen	AM8552
Ethidium bromide	Sigma-Aldrich	E1510
10× NorthernMax MOPS Gel Running Buffer	Invitrogen	AM8671
37% formaldehyde	Merck	104002
AMPure XP SPRI beads	Beckman	A63882
dNTP mix, 10 mM	Thermo Scientific	R0193
10 X T4 DNA Ligation Buffer	NEB	B0202S
Fast DNA End Repair Kit	Thermo Fisher	K0771
Buffer for Taq polymerase, 10× (Mg2 + free)	Invitrogen	P/N Y02028
Taq polymerase (non-hot start) (Native)	Thermo	18038042
MgCl2, 50 mM	Invitrogen	P/N Y02016
Platinum Taq polymerase, 5 U/μL	Invitrogen	P/N 100021273
TMAC (tetramethylammonium chloride) buffer, 5M	Sigma Aldrich	T3411-500ML
Fast DNA End Repair Kit	Thermo Fisher	K0771
RPMI 1640 Medium, GlutaMAX Supplement, HEPES	Gibco	72400054
Gibco Trypsin-EDTA (0.05%), Phenol red (500 mL)	Thermo Fischer	21985023
Heat-inactivated FBS	Thermo Scientific	10500064
Penicilin/Streptomycin 10 000 U/mL, 100 mL	Gibco/Thermo	15140122
TE buffer	Life Technologies	AM9849

**Oligonucleotides**		

Probe for GUIDE-seq dsODN: dsODN probe-fwd: /5HEX/TT + G + A + G+TTG+T+CATATGT/3IABkFQ/ and dsODN probe-rev: /5HEX/ACATATG+A+CAA+C+T + C + AA/3IABkFQ/	This protocol	N/A
dsODN for GUIDE-seq 5ʹ- P-G∗T∗TTAATTGAGTTGTCATATG TTAATAACGGT∗A∗T -3ʹ 5ʹ- P-A∗T∗ACCGTTATTAACATATG ACAACTCAATTAA∗A∗C -3ʹ	GUIDE-seq paper Tsai et al.^2^	N/A
MiSeq Common Adapter_MI [Phos]GATCGGAAGAGC∗C∗A	GUIDE-seq paper Tsai et al.^2^	N/A
A01 AATGATACGGCGACCACCGAGATCTAC ACTAGATCGCNNWNNWNNACACTCT TTCCCTACACGACGCTCTTCCGATC∗T	GUIDE-seq paper Tsai et al.^2^	N/A
A02 AATGATACGGCGACCACCGAGATCTACACCTCTCT ATNNWNNWNNACACTCTTTCCCTACACGACGCT CTTCCGATC∗T	GUIDE-seq paper Tsai et al.^2^	N/A
A03 AATGATACGGCGACCACCGAGATCTACACTAT CCTCTNNWNNWNNACACTCTTTCCCTACACGAC GCTCTTCCGATC∗T	GUIDE-seq paper Tsai et al.^2^	N/A
A04 AATGATACGGCGACCACCGAGATCTACACAG AGTAGANNWNNWNNACACTCTTTCCCTACACG ACGCTCTTCCGATC∗T	GUIDE-seq paper Tsai et al.^2^	N/A
A05 AATGATACGGCGACCACCGAGATCTACAC GTAAGGAGNNWNNWNNACACTCTTTCCCTACA CGACGCTCTTCCGATC∗T	GUIDE-seq paper Tsai et al.^2^	N/A
A06 AATGATACGGCGACCACCGAGATCTACACA CTGCATANNWNNWNNACACTCTTTCCCTACAC GACGCTCTTCCGATC∗T	GUIDE-seq paper Tsai et al.^2^	N/A
A07 AATGATACGGCGACCACCGAGATCTACA CAAGGAGTANNWNNWNNACACTCTTTCCCTA CACGACGCTCTTCCGATC∗T	GUIDE-seq paper Tsai et al.^2^	N/A
A08 AATGATACGGCGACCACCGAGATCTACAC CTAAGCCTNNWNNWNNACACTCTTTCCCTAC ACGACGCTCTTCCGATC∗T	GUIDE-seq paper Tsai et al.^2^	N/A
A09 AATGATACGGCGACCACCGAGATCTACACG ACATTGTNNWNNWNNACACTCTTTCCCTACA CGACGCTCTTCCGATC∗T	GUIDE-seq paper Tsai et al.^2^	N/A
A10 AATGATACGGCGACCACCGAGATCTACACA CTGATGGNNWNNWNNACACTCTTTCCCTACA CGACGCTCTTCCGATC∗T	GUIDE-seq paper^2^	N/A
A11 AATGATACGGCGACCACCGAGATCTACAC GTACCTAGNNWNNWNNACACTCTTTCCCTA CACGACGCTCTTCCGATC∗T	GUIDE-seq paper Tsai et al.^2^	N/A
A12 AATGATACGGCGACCACCGAGATCTAC ACCAGAGCTANNWNNWNNACACTCTTTCCCT ACACGACGCTCTTCCGATC∗T	GUIDE-seq paper Tsai et al.^2^	N/A
A13 AATGATACGGCGACCACCGAGATCTAC ACCATAGTGANNWNNWNNACACTCTTTCCC TACACGACGCTCTTCCGATC∗T	GUIDE-seq paper Tsai et al.^2^	N/A
A14 AATGATACGGCGACCACCGAGATCTACA CTACCTAGTNNWNNWNNACACTCTTTCCCT ACACGACGCTCTTCCGATC∗T	GUIDE-seq paper Tsai et al.^2^	N/A
A15 AATGATACGGCGACCACCGAGATCTACA CCGCGATATNNWNNWNNACACTCTTTCCC TACACGACGCTCTTCCGATC∗T	GUIDE-seq paper Tsai et al.^2^	N/A
A16 AATGATACGGCGACCACCGAGATCTACA CTGGATTGTNNWNNWNNACACTCTTTCCC TACACGACGCTCTTCCGATC∗T	GUIDE-seq paper Tsai et al.^2^	N/A
P701 CAAGCAGAAGACGGCATACGAGATTCGC CTTAGTGACTGGAGTCCTCTCTATGGGCA GTCGGTGA	GUIDE-seq paper Tsai et al.^2^	N/A
P702 CAAGCAGAAGACGGCATACGAGATCTAGT ACGGTGACTGGAGTCCTCTCTATGGGCAGT CGGTGA	GUIDE-seq paper Tsai et al.^2^	N/A
P703 CAAGCAGAAGACGGCATACGAGATTTCTGC CTGTGACTGGAGTCCTCTCTATGGGCAGT CGGTGA	GUIDE-seq paper Tsai et al.^2^	N/A
P704 CAAGCAGAAGACGGCATACGAGATGCTCA GGAGTGACTGGAGTCCTCTCTATGGGCAGT CGGTGA	GUIDE-seq paper Tsai et al.^2^	N/A
P705 CAAGCAGAAGACGGCATACGAGATAGGAGT CCGTGACTGGAGTCCTCTCTATGGGCAGT CGGTGA	GUIDE-seq paper Tsai et al.^2^	N/A
P706 CAAGCAGAAGACGGCATACGAGATCATGCC TAGTGACTGGAGTCCTCTCTATGGGCAGTCGG TGA	GUIDE-seq paper Tsai et al.^2^	N/A
P707 CAAGCAGAAGACGGCATACGAGATGTAG AGAGGTGACTGGAGTCCTCTCTATGGGCAGT CGGTGA	GUIDE-seq paper Tsai et al.^2^	N/A
P708 CAAGCAGAAGACGGCATACGAGATCCTCT CTGGTGACTGGAGTCCTCTCTATGGGCAGTC GGTGA	GUIDE-seq paper Tsai et al.^2^	N/A
P5_1 AATGATACGGCGACCACCGAGATCTA	GUIDE-seq paper Tsai et al.^2^	N/A
P5_2 AATGATACGGCGACCACCGAGATCTACAC	GUIDE-seq paper Tsai et al.^2^	N/A
Index1 ATCACCGACTGCCCATAGAGAGGACTCCAGTCAC	GUIDE-seq paper Tsai et al.^2^	N/A
Read2 GTGACTGGAGTCCTCTCTATGGGCAGTCGGTGAT	GUIDE-seq paper Tsai et al.^2^	N/A
Nuclease_off_+_GSP1 GGATCTCGACGCTCTCCCTATACCG TTATTAACATATGACA	GUIDE-seq paper Tsai et al.^2^	N/A
Nuclease_off_-_GSP1 GGATCTCGACGCTCTCCCTGTTTA ATTGAGTTGTCATATGTTAATAAC	GUIDE-seq paper Tsai et al.^2^	N/A
Nuclease_off_+_GSP2 CCTCTCTATGGGCAGTCGGTGAT ACATATGACAACTCAATTAAAC	GUIDE-seq paper Tsai et al.^2^	N/A
Nuclease_off_-_GSP2 CCTCTCTATGGGCAGTCGGTGATTTGA GTTGTCATATGTTAATAACGGTA	GUIDE-seq paper Tsai et al.^2^	N/A

**Other**		

Vortex	N/A	N/A
Bioruptor pico microtubes	Diagenode	Cat#C30010016
Bioruptor pico sonicator device	Diagenode	Cat#B01060010
Bioanalyzer	Agilent	Cat#G2939B
Cooled micro centrifuge for 1.5–2.0 mL tubes	N/A	N/A
Horizontal electrophoresis system and basic power supply	N/A	N/A
Computer-controlled CCD camera system with a UV transilluminator	Bio-Rad ChemiDoc Image System	12003153
Agarose gel electrophoresis system (chamber, combs etc.)	Bio-Rad, Wide Mini-Sub Cell GT Horizontal Electrophoresis System	1640301
Lonza 4D-Nucleofector Core Unit	Lonza	AAF-1003B
Lonza 4D-Nucleofector X Unit	Lonza	AAF-1003X
Lonza 4D-Nucleofector 96-well Unit	Lonza	AAF-1003S
Countess II Automated Cell Counter	Invitrogen	AMQAX1000
Nanodrop	Thermo	Nanodrop 2000
Qubit	Thermo	Qubit 3.0

## Materials and equipment

***Alternatives:*** In this protocol we use a Bioanalyzer to assess the extent of DNA fragmentation for GUIDE-seq. Alternatives are for example the TapeStation (Agilent) or LabChip (PerkinElmer).

The use of a Bioanalyzer (or equivalent) is preferred over an agarose gel since the Bioanalyzer has a better resolution in assessing the exact fragmentation pattern. An agarose gel can distinguish large differences in shearing (500–1000 bp fragments vs. 200–300 bp fragments), but with an agarose gel it is hard to distinguish between more subtle differences (200–300 bp fragments vs. 300–400 bp fragments). In addition, the Bioanalyzer (or equivalent) is also more sensitive and only requires a small fraction of material compared to what is needed to visualize fragmentation using an agarose gel. At the same time, the Bioanalyzer can detect adapter dimers contamination in sequencing final libraries.

***Alternatives:*** This protocol we used Bioruptor to sonicate DNA for GUIDE-seq. Alternatives are other brand of ultrasonicator, for example Covaris Focused-ultrasonicators etc.

***Alternatives:*** In this protocol we used ddPCR (digital droplet PCR) to detect the editing results (HDR and NHEJ editing) and the GUIDE-seq dsODN integration rates. (Refer to https://www.bio-rad.com/en-no/product/ddpcr-genome-edit-detection-assays?ID=PG9ALWE08O1Y for the design and testing of the probes for ddPCR). Different cell types can have different patterns in ddPCR readouts, while the qPCR method can be used as well.^3^^,^^4^

***Note:*** The editing results from ddPCR should be confirmed with amplicon sequencing during optimization. From our experience, generally, HDR results correlate very well, whereas NHEJ is often overestimated; however, if the same gating is used in all samples the NHEJ ratios will be correct.

**Table:** Full cell culture medium

Reagent	Final concentration	Amount
RPMI 1640 Medium, GlutaMAX Supplement, HEPES	N/A	500 mL
Heat inactivated FBS	10%	50 mL
Penicilin. Streptomycin 10 000U/mL	1%	5 mL
**Total**	**N/A**	**555 mL**

Store at 4°C. Right before use, water bath or waterless beads bath heat to 37°C.

***Note:*** Full cell culture medium used for cells just after the electroporation should not contain any antibiotics (Penicillin, Streptomycin).

**Table:** Electroporation Buffer

Reagent	Final concentration	Amount
KCl	5 mM	0.37 g
MgCl_2_ x 6H_2_O	15 mM	3.04 g
Phosphate buffer∗ consisting of: Na_2_HPO_4_ × 7H_2_O and NaH_2_PO_4_ × 2H_2_O	120 mM 80mM 40mM	21.42 g 6.24 g
D-mannitol	50 mM	9.11 g
Milli Q water	N/A	To 1 L
**Total**

***Note:*** Place all the weighted chemicals, except D-mannitol, in the same bottle. Add 800 mL of Milli Q water. Add clean, magnetic stirring bar and place the bottle on a magnetic stirrer to dissolve all chemicals. Measure pH and adjust it to pH 7.2 when all components are visibly dissolved. Add D-mannitol and magnetic stir for 10 min. Add water to 1 L and mix everything well. Filter sterilized the buffer. Prepare aliquots and Store at −20°C for 1 year.

***Note:*** ∗ Phosphate Buffer (pH 5.8 to 7.4) Preparation and Recipe can be found here: Phosphate Buffer (pH 5.8 to 7.4) Preparation and Recipe | AAT Bioquest

## Step-by-step method details

### *In vitro* transcription of Cas9 mRNA – Day 1

**Timing: 8 h**

Cas9 expression plasmid was linearized and Cas9 mRNA was *in vitro* transcribed, purified and then quality inspected by electrophoresis.

#### Plasmid linearization

1. Check the map of plasmid which will be used as the DNA template and chose proper digestion enzyme to linearize this plasmid. Any plasmid expressing the Cas9 or Cas9-fusion with a T7 promoter can be used, such as AddGene plasmid #101178 , #183194 etc.
2. Based the chosen enzyme, determining the information below:
  a. recommended digestion buffer.
  b. recommended plasmid amount (optimally: 8000 ng).
  c. recommended reaction incubation time and temperature.
3. Run the digestion reaction according to the information obtained from above (Step-2).

***Note:*** Here is an example reaction for using **MssI** to digest Cas9wt plasmid

**Table:** 

Reagent	Amount
Cas9wt plasmid	8 μg (× μL)
FastDigest MssI enzyme (Thermo Scientific, FD1344)	2 μL
10x digestion buffer	2 μL
H2O	to 20 μL
**Total**	**20 μL**

Reaction Condition: incubate at 37°C, 15 min

4. Run agarose gel to check linearization quality and product size.

#### mRNA *in vitro* transcription

5. Thaw the necessary kit (HiScribe T7 ARCA mRNA Kit (with tailing)) components, mix and pulse-spin in microfuge to collect solutions to the bottoms of tubes.
6. For each linearized plasmid, take 1μg and assemble the reaction in the following order in a 1.5 mL Eppendorf tube:

**Table:** 

Reagent	Amount
Nuclease-free water	to 20 μL
2× ARCA/NTP Mix	10 μL
Template DNA (linearized plasmid)	X μl (1 μg)
T7 RNA Polymerase Mix	2 μL
**Total**	**20 μL**

7. Mix thoroughly and pulse-spin in a microfuge. Incubate at 37°C on heat block for 30 min.

**Pause point:** the reaction can be stored at –20°C for one week.

***Note:*** Reaction time depends on template amount, quality, and RNA transcript length. For reactions with transcripts longer than 0.5 kb, 30 min incubation should give the maximum yield.

8. To remove the DNA template, add 2 μL of DNase I to each tube from pervious step (step 7). Mix well and incubate at 37°C for 15 min.

***Note:*** Keep the DNase I on the ice or in the freezing rack.

**Optional:** If desired, save 1 μL for gel analysis.

***Note:*** Do not heat the reaction or purify the RNA in this step and continue to proceed to the tailing reaction (step 9).

9. The Poly(A) tailing reaction can be set up as below in each tube from the previous step (step 8):

**Table:** 

Reagent	Amount
H_2_O	to 20 μL
IVT reaction (from previous step)	20 μL
10× Poly(A) Polymerase Reaction Buffer	5 μL
Poly(A) Polymerase	5 μL
**Total**	**50 μL**

***Note:*** Keep the PolyA polymerase on the ice or in the freezing rack.

***Note:*** The unpurified IVT reaction contains enough ATP, no extra ATP is necessary for the tailing reaction. Standard tailing reaction volume is 50 μL.

10. Mix thoroughly and pulse-spin in a microfuge. Incubate at 37°C for 30 min. Save 1 μL for analysis if necessary.

#### mRNA purification by LiCl precipitation

11. Add 25 μL of LiCl solution (7.5 M Lithium Chloride, 50 mM EDTA) to the 50 μL transcription reaction, and mix well. The final concentration of LiCl in the RNA solution is 2.5 M.
12. Incubate at –20°C for 30 min or longer. If needed, keep it overnight at –20°C.
13. Centrifuge at 4°C for 15 min at top speed (>12000rpm) of a microcentrifuge to pellet the RNA.
14. Remove the supernatant carefully. Rinse the pellet by slowly adding 500 μL of ice-cold 70% ethanol to remove residual salt, and then centrifuge at 4°C for 10 min.
15. Remove the ethanol carefully. Spin the tube briefly to bring down any liquid on the wall. Remove any residual liquid carefully with a sharp pipette tip (e.g., 10μL).
16. Air dry the pellet – a white or transparent pellet of the RNA will form within few minutes (do not dry more than 10 min).
17. Resuspend the mRNA in nuclease-free water. Make aliquots in PCR tubes, label and store the mRNA at –20°C (less than 6 months) or –80°C (more than 6 months).

**Pause point:** the reaction can be stored at –20°C for a short time before run mRNA electrophoresis.

#### mRNA electrophoresis

This is a quality control step for the *in vitro* transcribed mRNA, in order to check if the size of the mRNA is correct, and if any mRNA degradation happened, thus this step is highly recommended. An RNA integrity number (RIN) with a Bioanalyzer is also recommended.

18. Prepare the denaturing gel:
  a. add 1 g agarose in 72 mL water and heat until dissolved, then cool to 60°C.
  b. In the fume hood, add 10 mL 10× MOPS running buffer and 18 mL 37% formaldehyde (12.3 M) to the agarose.
  c. Mix gently but thoroughly by inverting the bottle several times. Do not introduce bubbles in the melted mixture.
    **CRITICAL:** Formaldehyde is toxic through skin contact and inhalation of vapors. Manipulations involving formaldehyde should be done in a chemical fume hood.
  d. In the fume hood: Pour the gel into the plastic chamber and insert the gel comb. Use a comb that will form wells large enough to accommodate at least 25 μL.
    ***Note:*** If you see any bubbles formed, remove then by poking them with a small pipette tip. Let the gel solidify for at least 2 h in the fume hood.
  e. Assemble the gel in the tank and add enough 1× MOPS running buffer to cover the gel.
    ***Note:*** Normally, the gel solidified in 30 min to 1 h. Do not let the gel dry out in the hood. Then remove the comb.
19. Prepare the mRNA sample:
  a. Add no more than 5 μL of the sample RNA to a 1.5-mL microcentrifuge tube. Add 3 volumes of Formaldehyde Load Dye to the sample RNA.
    ***Note:*** Each tube should typically contain 0.5–2 μg of poly(A)+ mRNA. If the volume of the sample RNA is larger than 5 μL, first precipitate the RNA and resuspend in a smaller volume.
  b. Incubate the samples for 15 min in a 65°C water bath to denature any RNA secondary structure. Briefly spin down samples in a microcentrifuge and place on ice.
  c. Add 0.5 μL ethidium bromide to the samples to visualize the RNA directly during and after electrophoresis.
    i. Load the samples on a denaturing formaldehyde agarose gel using RNase-free pipette tips.
      ***Note:*** To keep the samples as dense as possible, make sure there is no air trapped in the end of the pipette tip.
    ii. Place the tip just inside the top of the well.
    iii. Expel the sample slowly.
    iv. Gently raise the tip out of the well.
      ***Note:*** Remember to record the well number and corresponding sample name.
      **CRITICAL:** Ethidium bromide is a mutagenic compound that intercalates double-stranded DNA and RNA. Wear protective gloves, clothing, and eye/face protection when handling.
      ***Note:*** Ethidium bromide can be replaced by SYBR Safe Stain from Invitrogen. SYBR Safe Stain is specifically formulated to be a less hazardous alternative to ethidium bromide and it can be viewed with blue-light or by UV excitation. The stain is also suitable for staining RNA in gels, and in our experience, when using SYBR Safe Stain, the resolution of the gel is slightly worse than using ethidium bromide.
20. Run the gel:
  a. Load the RNA size markers on the gel and run gel with 10V voltage per centimeter of distance between electrodes.
    ***Note:*** In general, stop electrophoresis when the bromophenol blue dye front (corresponding to approximately 500 nt) has migrated approximately 3/4 the length of the gel. (Usually 1–2 h)
  b. Use a computer-controlled CCD camera system with a UV transilluminator to photograph the gel.
    ***Note:*** Any gel imaging system with ultraviolet (UV) light between wavelength 300 and 360 nm can be used to photograph the gel, for the ethidium bromide has UV absorbance maxima at this wavelength range.
    **CRITICAL:** Agarose gel containing formaldehyde are always handled and run in the chemical hood

### Editing fibroblast with Cas9 mRNA electroporation transfection – Day 2–6

**Timing: 4–5 h**

Cultured fibroblasts are electroporated with *in vitro* transcribed Cas9 mRNA and other related editing components - sgRNA and ssDNA repair template.

***Note:*** Transfection results may be donor dependent. Culture conditions may differ between cell types. Please follow your established procedure or the supplier’s recommendations.

***Note:*** It's critical to use sgRNA, not crRNA-tracrRNA hybrids as the latter won't work.

***Note:*** The average HDR editing levels that can be reached with the protocol and using of CleanCap mRNA gives somewhat higher editing levels. (Please refer to https://www.trilinkbiotech.com/cleancap for more information about “CleanCap mRNA Capping Technology”)

#### Pre-program the electroporation unit

21. Turn on the Core Unit of Lonza 4D-Nucleofector, follow the instruction of the instrument, select the wells will be electroporated and set the “Solution”, “Plus Code” accordingly, then leave the instrument at this state for now.

***Note:*** Depends on the number of the sample/replicates, 16 well stripe or 96 well electroporation plate can be used.

#### Electroporation of the fibroblasts

22. Check the cells for confluency. The cells shall reach 75%–80% of confluency before they can be subjected to electroporation.
23. Aspirate cell culture medium in T-175 flask and wash cells once with 35mL pre-warmed PBS.

***Note:*** for other sizes of dishes and flasks used for cell culture, some useful numbers can be found from https://www.thermofisher.com/no/en/home/references/gibco-cell-culture-basics/cell-culture-protocols/cell-culture-useful-numbers.html

24. Aspirate PBS and add 15mL pre-warmed Trypsin into the flask. Incubate at 37°C for 5 min until all cells have detached. Gently tap the flask and confirm proper dissociation using a microscope.
25. Add pre-warmed cell culture medium up to 30mL and pipette up and down using a 25mL pipette. Check under microscope for cell presence.
26. Transfer cell suspension into a 50mL Falcon tube and centrifuge at 300×g (relative centrifugal force, RCF) for 5min.
27. Discard supernatant and first resuspend cell pellet in 1000μL pre-warmed cell culture medium using a 1000μL pipette, then fill up to 10mL with pre-warmed cell culture medium. Take an aliquot for counting.
  a. Mix 10 μL Trypan Blue+10 μL cells and count with Countess II. Record cell viability and amount of living cells/mL.

***Note:*** Be as gentle as possible with cells and avoid vigorous pipetting.

28. Take the required volume of the cells for electroporation.
29. Prepare mRNAs, sgRNA and ssDNA repair template and Cas9WT RNP in 96-well PCR plate.
  a. Take out the Cas9 RNAs out from −80°C (or –20°C) and place it on ice.
  b. Take out the sgRNA(s) and ssDNA repair template(s) and place on ice. Let thaw completely.
  c. Place a sterile 96-well plate into the hood and pipette 1 μL of target gene sgRNA (100 μM) into each needed well for electroporation (for the test group(s) and control group(s)).
  d. Next, pipette 1 μL of IDT Cas9WT protein (60 μM) (used for positive control) into the wells (wells already added sgRNA from Step 29-c) going to be used for electroporation to form RNPs.
    i. Cover the plate with tinfoil, briefly spin, and mix gently with a pen scrapping over the bottom of the 96-well plate.
    ii. Place into the bench at room temperature (around 20°C–30°C) and let incubate for 15 min.
    iii. After that, place on ice.
      ***Note:*** Prepare Cas9WT RNP wells as positive control at this step.
  e. Then, add 1 μL of ssODN repair template (100μM) for the editing locus into each well containing RNPs (positive control) or containing sgRNA only (test group, will add Cas9 in mRNA format later). Cover with foil again, place on ice until use.
    ***Note:*** mRNA will be added later to avoid degradation.
30. Spin the cells for 5 min at 300g. Remove supernatant, add 30mL of warm PBS, wash gently and spin once more for 5 min at 300g. Remove the PBS and resuspend in an appropriate volume of electroporation buffer (20μL/well). Resuspend gently, but thoroughly.
31. Pipette 20μL of cell suspension in electroporation buffer onto the plate containing sgRNA, RNPs and ssODNs. Mix briefly. Keep the plate on a cooling rack (4°C).
32. Pipette the mRNAs onto corresponding test wells at this step. Use 100μL pipette mix once before loading into the electroporation strip.

***Note:*** Use ethanol and RNase Away spray to clean the hood environment to sure sterility of the mRNA.

33. Load 20μL into corresponding wells of the electroporation strip(s)/plate(s). Check for the bubbles, gently tap with the strip(s)/plate(s) on the bench or palm of hand and then proceed with electroporation.
34. Check the Lonza machine to make sure the “Solution”, “Plus Code” are set correct. Press “START” and perform electroporation.
35. Transfer the electroporation strip(s)/plate(s) back to the hood, then add 80μL of pre-warmed full cell culture media (without Penicilin and Streptomycin, P&S) into each strip(s)/plate(s) well. Place the electroporation strip(s)/plate(s) into the incubator for 15 min.
36. Prepare the cell culture plate(s)/dishes with full cell culture medium (without P&S).

***Note:*** full cell culture medium without antibiotics (P&S) is used for cells just after the electroporation to avoid excessive cell death.

37. After 15 min, place back the strip(s)/plate(s) into the bench. Distribute the cell suspension from electroporation strip(s)/plate(s) into corresponding wells of the cell culture plate(s). Make a crisscross movement with the culture plate(s) to ensure equal distribution of cells.
38. Check the cell culture plate(s) under the microscope and place into the incubator.
39. Next day, remove old media from previous day and add warm one with the P/S.
40. And harvest sample 4 days after the electroporation (if we count electroporation day is day-1, then harvest day is day-5), then extracted genome DNA.

### Profile off-target editing by optimized GUIDE-seq method – Day 2 and Day7–10

**Timing: 4–5 h (Day 2) + 4 days**

Use dsODN probes and ddPCR to find the optimal concentration of dsODN used for GUIDE-seq, and profile the off-target editing with the GUIDE-seq method.

***Note:*** Off-target is a common side effect from gene editing. GUIDE-seq (genome-wide, unbiased identification of DSBs enabled by sequencing) provides us with a genome-wide, unbiased, *in vitro* detection method to detect the off-target effect of living cells by sequencing.

***Note:*** an optimized GUIDE-seq method can be used in various cell types, such as primary human T cells including patient-specific variants, and is capable of identifying off-target cleavage/gene editing sites in a highly sensitive, unbiased, and genome-wide manner.

41. Optimize the dsODN concentration.
  ***Note:*** GUIDE-seq requires the transfection of double-stranded oligodeoxynucleotide (dsODN) into cells. Considering that different cell types have different tolerances to dsODN, for example, high concentration of dsODN are required for certain transfection refractory cells, and low concentration are needed for many primary cells sensitive to cytotoxic from the dsODN, therefore, the concentration of dsODN must be optimized according to the cell type.
  ***Note:*** The blunt-ended dsODN used in our GUIDE-seq experiments was the same as used in the original GUIDE-seq paper (Tsai et al., 2015), and prepared by annealing (in the same way as annealing the Y adapter) two modified oligonucleotides of the following compositions:
  5ʹ- P-G∗T∗TTAATTGAGTTGTCATATGTTAATAACGGT∗A∗T -3ʹ and,
  5ʹ- P-A∗T∗ACCGTTATTAACATATGACAACTCAATTAA∗A∗C -3ʹ.
  (Where P represents a 5ʹ phosphorylation and ∗ indicates a phosphorothioate linkage.)
  ***Note:*** To optimize the dsODN concentration, two Affinity Plus (IDT) probes were designed for dsODN used in GUIDE-seq, and the sequences of the two probes are:
  dsODN probe-fwd: /5HEX/TT + G + A + G+TTG+T+CATATGT/3IABkFQ/ and,
  dsODN probe-rev: /5HEX/ACATATG+A+CAA+C+T + C + AA/3IABkFQ/,
  These two probes use “HEX” as the 5′ reporter dye and “Iowa Black FQ” as the 3′ Quencher. And “+” stands for locked nucleic acids which impart heightened structural stability, leading to increased hybridization melt temperature (Tm) of these two relatively short probes that targeted the central part of dsODN. Both forward and reverse probes work well in the ddPCR experiments.
  a. The dsODN concentration optimization steps start with testing the different amounts of dsODN in cells undergoing CRISPR-Cas9 editing.
    ***Note:*** For optimization of dsODN concentration, both the amount of dsODN added and the volume of the electroporation transfection buffer system need to be considered. In our experiment setting, the proper concentration of dsODN after the test is 1 μM (20pmol in 20 μL electroporation transfection buffer), while in the original paper [Tsai, S. et al. 2015], the concentration of dsODN used in U2OS cells is 5 μM (100pmol in 20 μL buffer), and in HEK293 cells is 0,25 μM (5pmol in 20 μL buffer).
    ***Note:*** first, the dsODN is provided at the amount ranging from about 1 pmol to about 10 nmol to the cells (the specific range depends on cell types) during electroporation (step 22–39), and the cell viability was monitored every day after the dsODN transfection until the samples are harvested.
  b. When editing fibroblast with Cas9 mRNA electroporation transfection (day-2), add the dsODN in wells which will be used as to check the off-target profile. dsODN should be added together with other reagents just before electroporation (step 32).
  c. A group of blank cells (blank control), a group of cells only with dsODN (background control) and a group of cells with editing but no dsODN (dsODN integration negative control) are included as three controls.
    ***Note:*** here is an example (Figure 2):
    **Figure 2:**
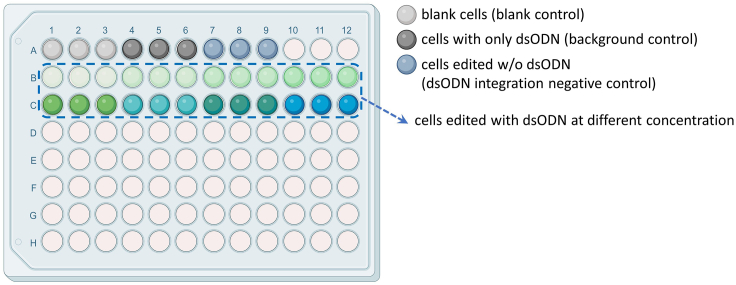
An example panel of experiment group design
  d. Next day, check the cell viability by taking aliquots for counting.
    Mix 10 μl Trypan Blue+10μl cells suspension and count with Countess. Record cell viability and amount of living cells/mL.
    ***Note:*** for Suspension cells, taking aliquots for counting is easy; and for adherent cells, cells need to be trypsinized into single cell suspension before counting, thus setting wells specifically be used for checking cell viability is strong recommended.
  e. Check and record cell viability in each condition every day until cells sample was harvested and draw cell viability curves accordingly.
  f. After sample harvested, the editing efficiency at that particular gene editing target is determined by ddPCR (according to the manual: *ddPCR* ™ *HDR Genome Edit Detection Assays*, 2017,https://www.bio-rad.com/sites/default/files/webroot/web/pdf/lsr/literature/10000065281.pdf, *ddPCR* ™ *NHEJ Genome Edit Detection Assays*, 2017 https://www.bio-rad.com/sites/default/files/webroot/web/pdf/lsr/literature/10000065278.pdf, or other paper^5^).
  g. At the same time, the dsODN integration rate to that particular target site is also determined by ddPCR with the dsODN probes mentioned above.
    ***Note:*** The dsODN integration rate at the target editing sites can be regarded as a representative parameter for all dsODN integration rates in whole genome double strands break sites for there is a direct relationship between them.
  h. After we obtained the cell viability (from step 40-d,e), target site editing efficiency and target site dsODN integration rates (from step 40-f), we can then comprehensively consider and determine the optimal dsODN concentration for a specific cell type under specific conditions.
42. GUIDE-seq sample preparation.
  a. Input Quantification and Shearing:
    i. Extracted genome DNA (dsDNA) is qualified by Nanodrop and quantified by Qubit.
      ***Note:*** when determining the sample by Nanodrop, the standard for a high-quality sample is as follows:
      260/280 (protein contamination) for pure DNA = 1.80, <1.80 ratio indicates protein contamination;
      260/230 (organic contaminants such as salts) for pure sample = 2, <1.8 indicates the presence of a significant organic contaminant.
    ii. In 0.65 mL Bioruptor Microtubes, gDNA is diluted to 100μL with 1× TE Buffer (DNA concentration is 1–20 ng/μL, and10 ng/μL is recommended);
    iii. Samples are vortexed (5–10 s) and centrifuged (10 s) before shearing; for optimal results, samples should be stored on ice during 5–10 min prior to sonication.
    iv. Each sample is sheared to an average length of 400–500 bp by using the “7 cycles, 15 s ON / 90 s OFF” condition according to the standard operating protocol for the Bioruptor Pico Sonicator at 4°C water cooler temperature.
      ***Optional:*** Run undiluted sheared gDNA samples (5 μL) with 1.2% agarose gel and image it to check the size and quality of gDNA.
    v. If the sheared gDNA size is right, take 400ng gDNA and brought to final volume of 120 μL using 1× TE Buffer.
    vi. a cleanup with 120 μL of AMPure XP SPRI beads (1× ratio) is performed according to manufacturer protocol (AMPure XP Instructions For Use, https://www.beckmancoulter.com/wsrportal/techdocs?docname=b37419) and eluted in 15μL of 1× TE Buffer.
  b. End Repair and A-tailing:
    i. To a 200 μL PCR tube or well in a 96-well plate, add the following (per reaction):

**Table:** 

Reagent	Amount
Nuclease-free H2O	1.5 μL
dNTP mix, 5mM	1.0 μL
10 X T4 DNA Ligation Buffer (NEB, B0202S)	2.5 μL
End-repair Enzyme mix	1.0 μL
Buffer for Taq Polymerase, 10× (Mg2 + free)	2.0 μL
Taq Polymerase (non-hot start) )	0.5 μL
**Total Volume**	**8.5 μL**
+ DNA sample (from previous step)	14.0 μL
**Total Volume**	**22.5 μL**

Mix well by brief vortex, and spin down, then put the tube/plate on thermcycler machine.

      ***Note:*** End-repair mix (low concentration) Enzymatics, Inc. in the original paper^2^ is not continue, we used End-repair Enzyme mix from “Fast DNA End Repair Kit” (Thermo Fisher, K0771) instead, and used less volume than original protocol.
      ***Note:*** Buffer for Taq Polymerase use here is Mg2+ free since the T4 DNA Ligation Buffer already contains the Mg2+.
    ii. Set the end repair thermocycler program as follow:

**Table:** 

Steps	Temperature	Time	Cycles
End Repair and A-tailing	12°C	15 min	1
	37°C	15 min	1
	72°C	15 min	1
Hold	4°C	forever
  c. Adapter Ligation:
    i. When the sample finished end-repair thermocycle program, to the sample reaction tube or well, add the following reagents in order (mix by pipetting) (per reaction):

**Table:** 

Reagent	Amount
Annealed Y adapter_MI (i5) (10μM)	1.0 μL
T4 DNA Ligase	2.0 μL
+ DNA sample (from previous step: 42-b-ii)	22.5 μL
**Total Volume**	**25.5 μL**

Then put the tube/plate on thermocycler machine.
    ii. Set the adapter ligation thermocycler program as follow:

**Table:** 

Steps	Temperature	Time	Cycles
Adapter Ligation	16°C	30 min	1
	22°C	30 min	1
Hold	4°C	forever
    iii. When adapter ligation finished, use 0.9× (22.95μL) Ampure XP SPRI beads to clean, elute in 12μL of 1× TE buffer.
      ***Optional:*** Take one control sample to run on agarose gel to check if the Y adapter_MI (78mer) was added. If it was added, there should be a shift of the DNA bands compared with the freshly sheared gDNA.
  d. PCR-1 (oligo tag primer [Discovery] or large primer pool [Deep-sequencing Validation]).
    i. Prepare the following PCR master mix (per reaction):

**Table:** 

Reagent	Amount
Nuclease-free H2O	11.9 μL
Buffer for Taq Polymerase, 10× (MgCl2 free)	3.0 μL
dNTP mix, 10 mM	0.6 μL
MgCl2, 50 mM	1.2 μL
Platinum Taq polymerase, 5 U/μL	0.3 μL
GSP1 Primer (10μM) / Primer Pool (∗)	1.0 μL∗
TMAC (0.5M)	1.5 μL
P5_1, 10 μM	0.5 μL
Total	20.0 μL
+ DNA sample (from Adapter Ligation Step 41-c-iii)	10.0 μL
**Total Volume**	**30.0 μL**

***Note:*** Discovery is to find novel off-target sites; validation is to confirm previously identified off-target sites.

∗ For Discovery, make separate master mixes for +/(sense) and –/(antisense) reactions, and proceed with separate PCR reactions. For deep-sequencing validation, one master mix can be made. Primer Pool should be normalized to a total amount of 30 pmol in the 30 μl reaction.
    ii. Discovery Thermocycler Program (touchdown) is set as follow:

**Table:** 

Steps	Temperature	Time	Cycles
Initial Denaturation	95°C	5 min	1
Denaturation	95°C	30 s	15 cycles
Annealing	70°C (−1°C/cycle)	2 min	
Extension	72°C	30 s	
Denaturation	95°C	30 s	10 cycles
Annealing	55°C	1 min	
Extension	72°C	30 s	
Final extension	72°C	5 min	1
Hold	4°C	forever
    iii. Or validation thermocycler program is set as follow:

**Table:** 

Steps	Temperature	Time	Cycles
Initial Denaturation	95°C	5 min	1
Denaturation	95°C	30 s	14 cycles
Annealing	20% ramping down to 65°C		
Extension	65°C	5 min	
Final extension	72°C	5 min	1
Hold	4°C	forever
    iv. After PCR, a cleanup with 1.2 X of AMPure XP SPRI beads (36.0 μL) is performed, elute in 15 μL of 1× TE Buffer.
  e. PCR-2 (oligo tag primer [Discovery] or large primer pool [Deep-sequencing Validation])
    i. Prepare the following PCR master mix (per reaction):

**Table:** 

Reagent	Amount
Nuclease-free H2O	5.4 μL
Buffer for Taq Polymerase, 10× (Mg2+ free)	3.0 μL
dNTP mix, 10mM	0.6 μL
MgCl2, 50 mM	1.2 μL
Platinum Taq polymerase, 5 U/μL	0.3 μL
GSP2 Primer (10μM)/Primer Pool (∗)	1.0 μL
TMAC (0.5 M)	1.5 μL
P5_2, 10 μM	0.5 μL
Total	13.5 μL
+ P7_# (10μM)∗	1.5 μL
+ DNA sample with beads (from step 41-d-ii)	15.0 μL
**Total Volume**	**30.0 μL**

      ***Note:*** ∗ Primer concentrations should follow the specifications described in PCR1.
      ***Note:*** For the P7_#, at least 4 should be used in one sequencing run for good image registration on Illumina sequencer (e.g., P701 – P704 or P705 – P708)
    ii. Discovery Thermocycler Program (touchdown): same as for PCR1.
    iii. Validation Thermocycler Program: same as for PCR1.
    iv. use 0.7× (21.0 μL) Ampure XP SPRI beads to clean, elute in 30μL of 1× TE buffer.
  f. Library quantification and send for sequencing.
    ***Note:*** Library quantification can be achieved by qPCR Quantification, such as using Kapa Biosystems kit for Illumina Library Quantification kit and conduced according to manufacturer instruction.

## Expected outcomes

A successful mRNA-delivered CRISPR editing should give a certain percentage of cells being edited. The actual editing efficiency varies. This depends on the cell types, edit genome locus, guide RNA used and some other unknown factors. Even over 50% editing efficiency can be reached for easy editing cells and locus, while for challenging editing cells, 1% or lower editing efficiency can be expected.

A successful GUIDE-seq Off-target check should identify the global DNA double-stranded breaks (DSBs) enabled by sequencing. It relies on capture of double-stranded oligodeoxynucleotides integrated into DSBs. Like the editing, GUIDE-seq results also vary on different cells and edit genome locus and guide RNA used.

## Quantification and statistical analysis

When optimize the concentration of dsODN for GUIDE-seq, we need to check the living cell number after the electroporation every day, and we can use the Table 1 as a template.

**Table 1:** Example of living cell number counting after Cas9 editing and dsODN electroporation (cell number unit: ×10^5^ cells/mL)

	2 pmol				5 pmol				10 pmol			
Day-1	3,23	3,87	2,87	3,17	3,23	3,46	2,52	3,58	3,23	2,76	2,93	1,88
Day-2	4,16	3,87	5,57	4,34	4,16	4,34	2,93	3,17	5,16	3,58	3,64	2,35
Day-3	3,58	4,57	4,05	4,87	2,11	3,58	2,58	1,82	2,93	3,17	2,58	1,88
Day-4	7,33	7,04	6,39	8,03	6,33	6,04	5,92	4,93	7,86	6,69	6,74	3,87
	20 pmol				50 pmol				100 pmol			
Day-1	3,05	1,99	2,87	2,29	2,05	2,25	1,99	1,99	2,05	1,58	2,52	1,88
Day-2	4,75	3,69	2,87	3,99	2,76	2,05	2,23	2,23	2,64	1,29	2,05	1,58
Day-3	4,16	3,93	2,17	2,52	1,35	1,47	1,94	1,29	1,11	0,938	0,821	0,887
Day-4	4,28	4,93	4,57	3,99	2,46	2,64	3,05	1,7	1,11	1,94	2,7	2,58
	200 pmol				500 pmol				w/o dsODN			
Day-1	1,7	2,29	2,82	2,46	1,41	2,17	1,88	1,76	3,58	2,76	3,52	2,46
Day-2	2,05	0,645	1,82	1,88	2,46	1,41	1,99	2,23	3,99	5,63	4,4	4,34
Day-3	0,762	1,17	0,88	1,99	1,17	0,997	1,29	1,94	4,34	3,58	4,22	5,16
Day-4	2,29	1,41	1,7	2,46	3,23	2,35	2,93	3,05	8,56	9,33	8,8	8,74
	Blank cells											
Day-1	2,4	3,69	1,88	1,82								
Day-2	3,64	5,34	2,52	2,29								
Day-3	3,81	4,52	3,11	2,35								
Day-4	5,69	11,3	7,62	6,28								

**Figure 3:**
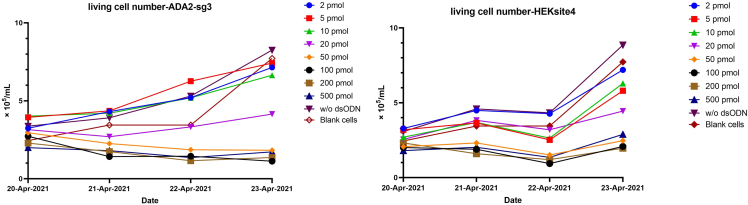
Living cell number after editing in two loci (Left: ADA2 site with single guide RNA #3 (sg-3). Right: HEKsite4 single guide RNA)

**Figure 4:**
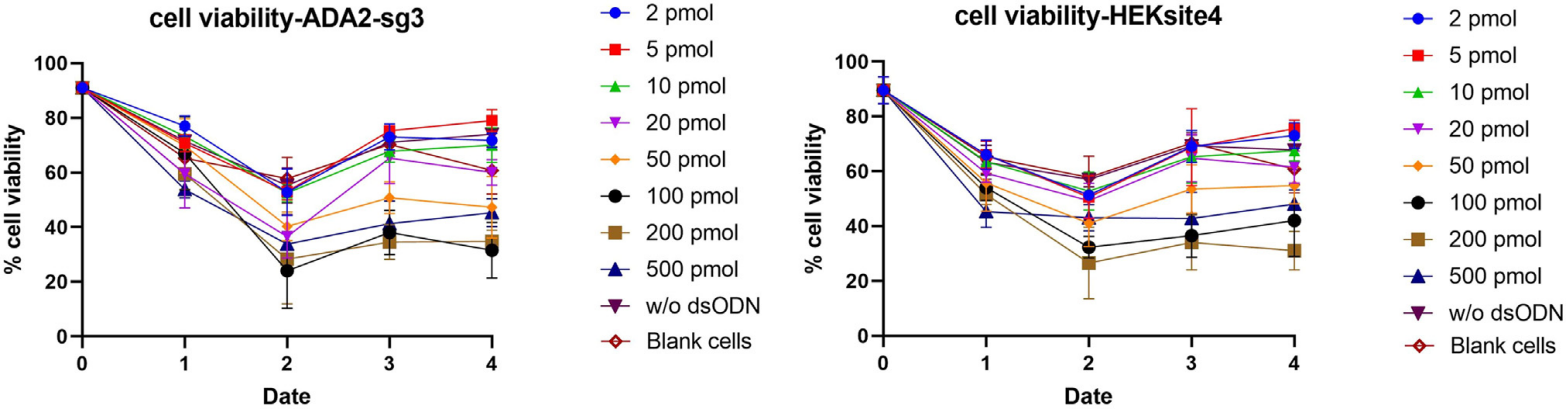
Cell viability after editing in two loci (Left: ADA2 site with single guide RNA #3 (sg-3). Right: HEKsite4 single guide RNA)

Based on Table 1 we draw line chart as follow (Figure 3):

We record the cell viability using the same way (Figure 4).

**Figure 5:**
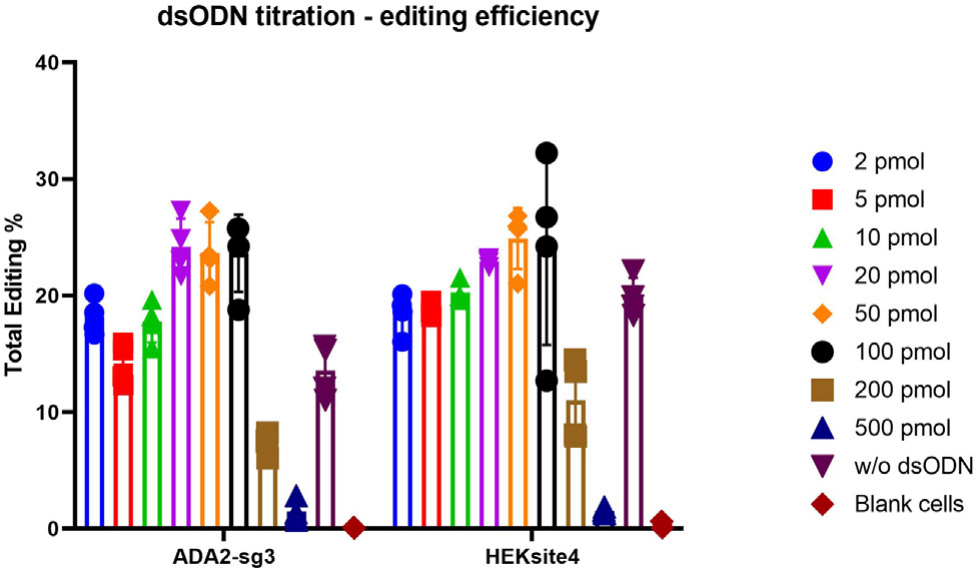
Editing efficiency when different dsODN amount used

**Figure 6:**
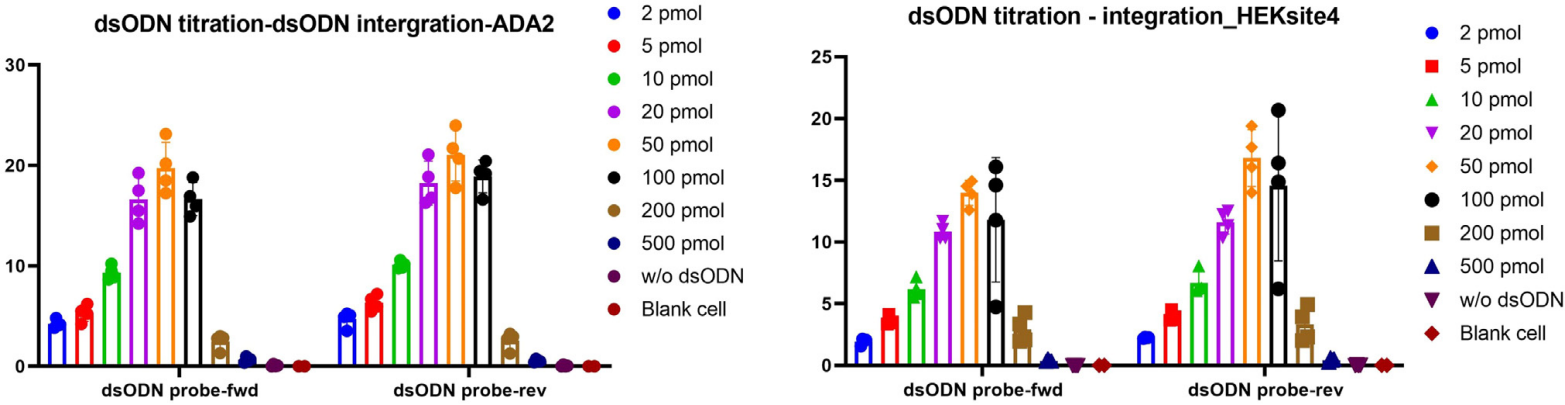
dsODN integration rate when different dsODN amount used (Left: ADA2 site with single guide RNA #3 (sg-3). Right: HEKsite4 single guide RNA)

Next, we check the editing efficiency and dsODN integration rate by ddPCR, then record the date in the similar way and draw the bar chart (Figures 5 and 6).

Comprehensively considerate these factors, the optimal dsODN concentration for a specific cell type under specific conditions can be determined. For example: in our experiment setting, first, we can see there is a significant decrease in living cell number and cell viability when the dsODN amount researched 50pmol; the editing efficiency increases alongside as the dsODN amount increased, and when using 20 pmol or 50 pmol dsODN, their editing efficiency is comparable; and dsODN integration rate for 50 pmol is slightly higher compared with 20 pmol. Thus, we can get the conclusion that the optimal dsODN concentration for our experiment setting should be in the range of 20–50 pmol dsODN added in 20μL electroporation transfection buffer. Finally, we determined to use 20 pmol in 20μL buffer (1 μM) as the final optimized dsODN concentration.

## Limitations

Although editing fibroblasts with *in vitro* transcribed Cas9 mRNA has several advantages, such as strong versatility, and strong scalability, it also has some room for improvement, like increasing the stability and decreasing immunogenicity. Large molecular weight of mRNA and low tolerance of chemical modifications that is necessary for deimmunogenicity and increased stability for the protein translation machinery make the development of mRNA drugs lag behind other nucleic acid drugs. mRNA itself is not a therapeutic molecule and needs to be "produced" in the body for function. Its pharmacokinetics are more complicated than those of monoclonal antibody drugs, and individual differences are greater, making it difficult to control.^6^

Electroporation can provide high transfection efficiency, but it requires an expensive instrument (electroporator). Also, it is greater damage to cells; meanwhile, it requires more cells and DNA per transfection. And the conditions for each type of cell electroporation require multiple optimizations.

In laboratory, when handling mRNA production, necessary measures to prevent RNA degradation should be taken, otherwise, degraded mRNA will not lead to effective editing results. When preparing the DNA sample for GUIDE-seq, the DNA quality should be good and the quantity should be suitable to avoid failure in the downstream library construction.

## Troubleshooting

### Problem 1

Low editing efficiency.

### Potential solution

If mRNA degradation happened, there will be not enough mRNA can be translated into Cas9 protein. This will lead to low editing efficiency. Thus, it is important to make sure the quality of mRNA is good. RNA integrity number (RIN) with Bioanalyzer is recommended.

Cells after many passages will not be suitable for electroporation, and this will also lead to low editing efficiency.

### Problem 2

Low DNA yield (step 39).

### Potential solution

If the cell lysis is sufficient, it will cause low DNA yields. Examining a small aliquot of cell lysate under a standard microscope (after step 39) can help us asset the extent of lysis.

### Problem 3

Failed GUIDE-seq library preparation (step 42).

### Potential solution

Failed GUIDE-seq library preparation means that when do library quantification, we cannot gain enough library for sequencing. Failed library preparation may be happened from failed adapter Ligation. Thus, to run an agarose gel (after step 42-c-iii) is recommended to check if the adapter was added. There should be a shift of the DNA bands compared with the freshly sheared gDNA If it was added.

## Resource availability

### Lead contact

Further information and requests for resources and reagents should be directed to and will be fulfilled by the lead contact, Emma Haapaniemi, e.m.haapaniemi@ncmm.uio.no.

### Materials availability

This study did not generate new unique reagents.

### Data and code availability

This study did not generate datasets/code.
